# Contribution of active surveillance cultures to the control of hospital-acquired carbapenem-resistant *Acinetobacter baumannii* in an endemic hospital setting

**DOI:** 10.1017/ice.2023.162

**Published:** 2024-02

**Authors:** Debby Ben-David, Yael Cohen, Gabrielle Levi, Alona Keren-Paz, Diana Tasher, Gisele Zandman-Goddard, Orna Schwartz, Yasmin Maor

**Affiliations:** 1 Wolfson Medical Center, Holon, Israel; 2 Sackler Faculty of Medicine, Tel Aviv University, Tel Aviv, Israel; 3 National Institute for Antibiotic Resistance and Infection Control, Israel Ministry of Health, Tel Aviv, Israel

## Abstract

**Background:**

Despite the increasing rates of carbapenem-resistant *Acinetobacter baumannii* (CRAB) carriage among hospitalized patients in endemic settings, the role of active surveillance cultures and cohorting is still debated. We sought to determine the long-term effect of a multifaceted infection-control intervention on the incidence of CRAB in an endemic setting.

**Methods:**

A prospective, quasi-experimental study was performed at a 670-bed, acute-care hospital. The study consisted of 4 phases. In phase I, basic infection control measures were used. In phase II, CRAB carriers were cohorted in a single ward with dedicated nursing and enhanced environmental cleaning. In phase III large-scale screening in high-risk units was implemented. Phase IV comprised a 15-month follow-up period.

**Results:**

During the baseline period, the mean incidence rate (IDR) of CRAB was 44 per 100,000 patient days (95% CI, 37.7–54.1). No significant decrease was observed during phase II (IDR, 40.8 per 100,000 patient days; 95% CI, 30.0–56.7; *P* = .97). During phase III, despite high compliance with control measures, ongoing transmission in several wards was observed and the mean IDR was 53.9 per 100,000 patient days (95% CI, 40.5–72.2; *P* = .55). In phase IV, following the implementation of large-scale screening, a significant decrease in the mean IDR was observed (25.8 per 100,000 patient days; 95% CI, 19.9–33.5; *P* = .03). An overall reduction of CRAB rate was observed between phase I and phase IV (rate ratio, 0.6; 95% CI, 0.4–0.9; *P* < .001).

**Conclusions:**

The comprehensive intervention that included intensiﬁed control measures with routine active screening cultures was effective in reducing the incidence of CRAB in an endemic hospital setting.

*Acinetobacter baumannii* has emerged in the 1970s as a major cause of hospital-acquired infections that cause ventilator-associated pneumonia, bloodstream infections, and wound infections.^1^ Over the past 20 years, *A. baumannii* has become resistant to many antibiotics, including carbapenems.^2^ Resistance rates vary widely between countries. In western Europe, carbapenem resistance among *A. baumannii* strains is <5%, compared with >50% in South Asia and Latin America.^3–5^ In a recent assessment of the global burden of antimicrobial resistance during 2019, *A. baumannii* was one of the leading pathogens responsible for >400,0000 deaths.^6^ The rapid global spread has driven the World Health Organization (WHO) to recognize carbapenem-resistant *A. baumannii* (CRAB) as the critical, number-one priority among a list of 12 antibiotic-resistant bacteria that pose the greatest threat to modern medicine.^7^

The use of contact precautions prevents the transmission of multidrug-resistant microorganisms (MDROs) and is considered the key component of control programs.^8^ However, unidentified colonized patients may serve as a potential reservoir for transmission of MDROs. In a recent study, the level of environmental contamination with CRAB was similar between carriers identified through surveillance cultures only and those with positive clinical cultures.^9^ The use of active screening cultures in combination with contact precautions was associated with persistent reductions in the incidence of carbapenemase-producing Enterobacteriaceae (CPE).^10–12^ Although some studies have included active screening programs as part of a multimodal approach to controlling CRAB infections in healthcare settings,^13,14^ the existing evidence is limited. As a result, the WHO and the European Society of Clinical Microbiology and Infectious Diseases (ESCMID) guidelines do not currently recommend active screening as a fundamental measure for preventing the spread of CRAB in healthcare settings.^7,15^

Since the early 2000s, CRAB strains have disseminated in several acute-care hospitals in Israel.^16^ Most infections were detected among inpatients in intensive care units (ICUs) and medical wards. Between 2014 and 2019, the national pooled mean percentage of carbapenem resistance among *A. baumannii* blood isolates reached 75%–80%.^17^ The primary aim of this study was to evaluate the impact of different components of a multifaced control program on the incidence of HA-CRAB in an endemic setting.

## Methods

### Hospital setting

Wolfson Medical Center is a 670-bed, secondary-care teaching hospital in central Israel. The hospital includes 4 ICUs and 6 medical wards. Each medical ward includes a step-up unit with 5 critical patients hospitalized in a multibed open room. The distance between the beds in each step-up unit is <1 m. The nurse-to-patient ratio during the study was 1:4.

### Study design

This study was a quasi-experimental intervention study based on open cohorts of patients admitted to the hospital from January 2019 to September 2022.

**Figure 1.**
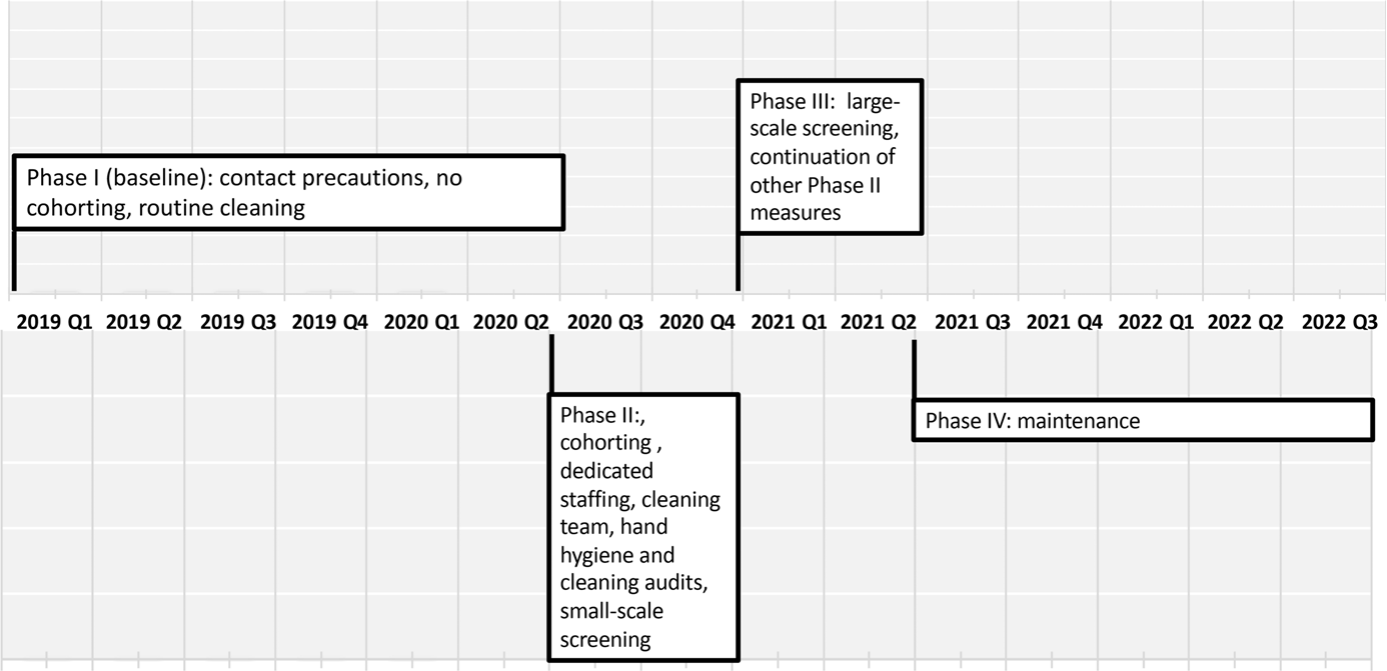
Timeline of prevention measures throughout the study period.

The study consisted of 4 phases: phase I - baseline (January 2019–May 2020); phase II - first intervention period (June 2020–December 2020); phase III - a 6-month second intervention period (January 2021– June 2021); phase IV - a 15-month follow-up period (June 2021–June 2022) (Fig. 1). In phase I, basic infection-control measures were used for CRAB-infected patients, including contact precautions. Patients were placed in separate rooms if available. Medical patients who required intensified care were isolated in the step-up unit. Detection of CRAB was based solely on clinical samples. Environmental cleaning was conducted by ward nurse aids using 1:1,000 sodium hypochlorite solution.

In phase II, all CRAB-infected patients were cohorted in a single ward and were managed by dedicated nurses. A dedicated team performed enhanced environmental cleaning, including terminal and daily cleaning. To assess cleaning quality, weekly fluorescent audits were conducted by infection preventionists and environmental services staff. Gel spots were placed on high-touch surfaces in patient rooms. A portable ultraviolet light was used to reevaluate rooms after cleaning; detection of residual fluorescence was a sign of inadequate cleaning. Cleaning staff received immediate and quarterly feedback for improvement. Hospital-wide overt hand hygiene audits with immediate feedback were conducted by infection control preventionists. We instituted small-scale screening by obtaining cultures from the roommates of patients who were newly detected with CRAB infection.

In phase III, despite high compliance with the interventions implemented in phase 2, during the early months of 2021, we observed CRAB dissemination in several wards. As a result, in addition to the measures begun in phase 2, we implemented large-scale screening. Patients transferred from long-term care facilities or with prior hospitalization within the previous 6 months were screened on admission to medical wards. All patients admitted to the 6 step-up units and adult ICUs were screened on admission and weekly. Screening sites included rectal, buccal, and respiratory secretions (from ventilated patients). To ascertain whether the outbreak was monoclonal, phenotypic characterization of all imported and hospital acquired CRAB isolates identified between January and March 2021, was conducted using Fourier transform infrared (FTIR) spectroscopy. Unfortunately, isolates from other phases were not available for FTIR spectroscopy analysis.

Phase IV comprised a follow-up period of 15 months. During this period, all interventions from the previous phases continued.

### Microbiological methods

Clinical isolates were identified by standard laboratory methods based on Clinical and Laboratory Standards Institute guidelines.^18^ Antibiotic susceptibility was determined using the disk-diffusion method. Surveillance samples were obtained using sterile swabs (Copan Italia S.p.a.) and were transported to the microbiology laboratory for the detection of CRAB. These swabs were streaked onto CHROMagar MDR *Acinetobacter* plates (Hylabs, Rehovot, Israel) and were incubated overnight at 37°C in ambient air. Suspicious colonies (ie, pink colonies) were identified to the species level using VITEK-MS (bioMérieux, Marcy l’Etoile, France). Carbapenem resistance was determined using meropenem (10 µg) disks.

### FTIR spectroscopy

FTIR spectroscopy was performed as previously described.^19^ Isolates were grown at 35±2°C on blood agar plates (HyLabs, Rehovot, Israel) for up to 24 hours. Samples were prepared according to the Infrared Biotyper (Bruker, Leipzig, Germany) according to the manufacturer’s instructions. We analyzed 4 replicates per sample, and average spectra were calculated using the Biotyper default settings. Spectra were analyzed using OPUS version 7.5 software (Bruker). Hierarchical cluster analysis (displayed as a dendrogram) was generated using OPUS software with the Pearson correlation coefficient option. We chose a cutoff value that defined a cluster by inspecting the resulting dendrogram (within the range 0.25–0.4 for *A. baumannii*, recommended by the manufacturer), which we believe represents the epidemiological distribution of our samples.

### Definitions

Patients with a positive clinical or screening culture during the first 3 days of hospitalization were defined as imported CRAB. Hospital-acquired clinical CRAB was defined as the isolation of the first clinical isolate after day 3 of hospitalization. A CRAB carrier was defined as a patient initially identified through surveillance cultures, either upon admission or by weekly screening. The efficacy of the program was evaluated through 2 quantifiable parameters: (1) the incidence density rate (IDR) of clinical HA-CRAB isolates (calculated per 100,000 patient days) and (2) the prevalence of newly detected CRAB carriage among patients who underwent screening cultures during hospitalization.

The clinical analysis included all patients with HA clinical isolates, regardless of whether they were identified through clinical cultures or initial screening followed by clinical cultures. This approach enabled a comparison of CRAB rates before and after the intervention.

Hand hygiene compliance was expressed as the percentage of correct actions over all observed opportunities. Compliance with environmental and medical equipment cleaning was assessed by determining the average percentage of high-touch surfaces that had been cleaned, as indicated by fluorescent markers.

### Statistical analyses

Differences in clinical HA-CRAB between periods were tested with ANOVA and post hoc Tuckey test for pairwise differences. The association between clinical HA-CRAB and compliance with various intervention measures was assessed using the Pearson correlation. To determine changes in the monthly proportion of clinical detections relative to total CRAB detections, linear regression analysis was performed. We used the χ^2^ test to separately assess the prevalence of CRAB positivity in patients who underwent weekly and admission screening in 2021 compared with 2022. All analyses were 2-tailed and were performed using SAS version 9.4 software (SAS Institute, Cary, NC). Significance was set at *P* < .05. The study was approved by the jurisdictional board review of the institution.

## Results

### Baseline phase

During phase I, 179 patients were newly detected through clinical microbiologic cultures, averaging 10.6 (SD ±4.5) per month. The average age of infected patients was 75 years (SD±16). Of all CRAB infections, 119 (66.5%) were hospital acquired, primarily in 6 medical wards and 2 adult ICUs, which accounted for 90.0% of cases. The median length of stay before detecting hospital-acquired acquisition was 13 days (IQR, 7–26). Following the clinical detection of CRAB, 55.8% of infected patients were isolated in the 6 step-up units for a median duration of 10 days (IQR, 4–7).

### Clinical HA-CRAB

**Figure 2.**
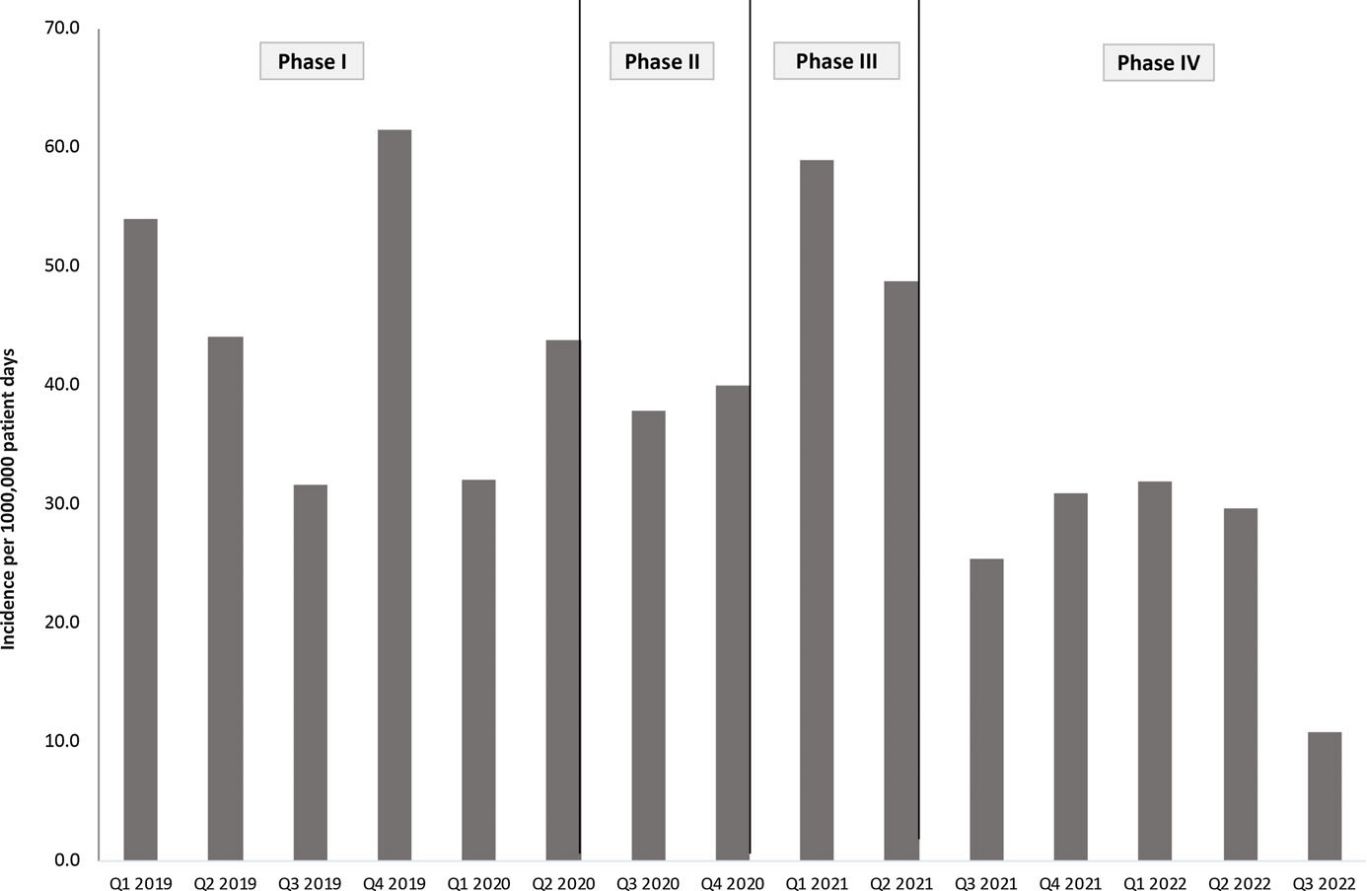
Incidence density of clinical hospital-acquired carbapenem-resistant *Acinetobacter baumannii* between 2019 and 2022. Note. Phase I (January 2019–May 2020), baseline measures; phase II (June–December 2020), cohorting CRAB carriers, dedicated staff, enhanced environmental cleaning, small-scale screening; phase III (January–June 2021), cohorting CRAB carriers, dedicated staff, enhanced environmental cleaning, large-scale screening; phase IV (July 2021–September 2022), follow-up.

During the study, we evaluated 45 consecutive months of data with 659,132 patient days. In total, 259 clinical HA-CRAB were identified. During the baseline period, the mean IDR of HA-CRAB was 44 per 100,000 patient days (95% CI, 37.7–54.1) No significant decrease was observed during phase II (IDR, 40.8 per 100,000 patient days; 95% CI, 30.0–56.7; *P* = .97). During phase III, ongoing transmission in several wards was observed, and the mean IDR increased to 53.9 per 100,000 patient days (95% CI, 40.5–72.2; *P* = .55). Following the implementation of active screening cultures, a significant decrease in the mean IDR (25.8 of 100,000 patient days; 95% CI, 19.9–33.5; *P* = .03) was observed. A significant overall reduction in clinical HA-CRAB rates was observed between phase I and phase IV (rate ratio, 0.6; 95% CI, 0.4–0.9; *P* < .001) (Fig. 2).

### Uptake of the interventions

In June 2020, a multifaceted intervention was introduced. The daily prevalence of hospitalized CRAB carriers was 6.5 (SD±2.6). The monthly average of cohorted patients with dedicated nursing was 87.8% (SD±9.5). In total, 1,338 high-touch points were marked in the rooms of CRAB patients. The monthly average of clean objects was 89.8% (SD±7.6). In total, 38,508 hand hygiene opportunities were conducted hospital-wide. Over time, the average monthly of clean high-touch objects did not change (Table 1). In contrast, a reduction was observed in hand hygiene compliance and in the monthly proportion of cohorted patients.

**Table 1.** Compliance With Control Measures

Phase	Months Observed	Cohorted Patients With Dedicated Nursing		Hand Hygiene		Environmental Cleaning	
		No. of Isolation Days	Cohorted % (±SD)	No. of Observations	Compliance % (±SD)	No. of High-Touch Points	Clean Sites % (±SD)
Phase 1	17	2,916	0	3,943^a^	77.5±3.2	0	NR
Phase 2	7	698	93.4±8.3	6,804	78.8±6.4	237	85.1±22.0
Phase 3	6	1,576	90.7±5.9	10,046	77.9±2.8	225	90.7±8.3
Phase 4	15	2,927	84.7±9.3	22,776	73.4±3.6	876	88.6±9.2
Estimate, % (*P* value)			−0.7 (0.04)		−1.4 (<.001)		−0.7 (0.14)

Note. CRAB, carbapenem-resistant *Acinetobacter baumannii*; NR, not relevant; SD, standard deviation.

a Hand hygiene observations by infection control preventionists were initiated in January 2020.

### Fourier-transform infrared (FTIR) spectroscopy

**Figure 3.**
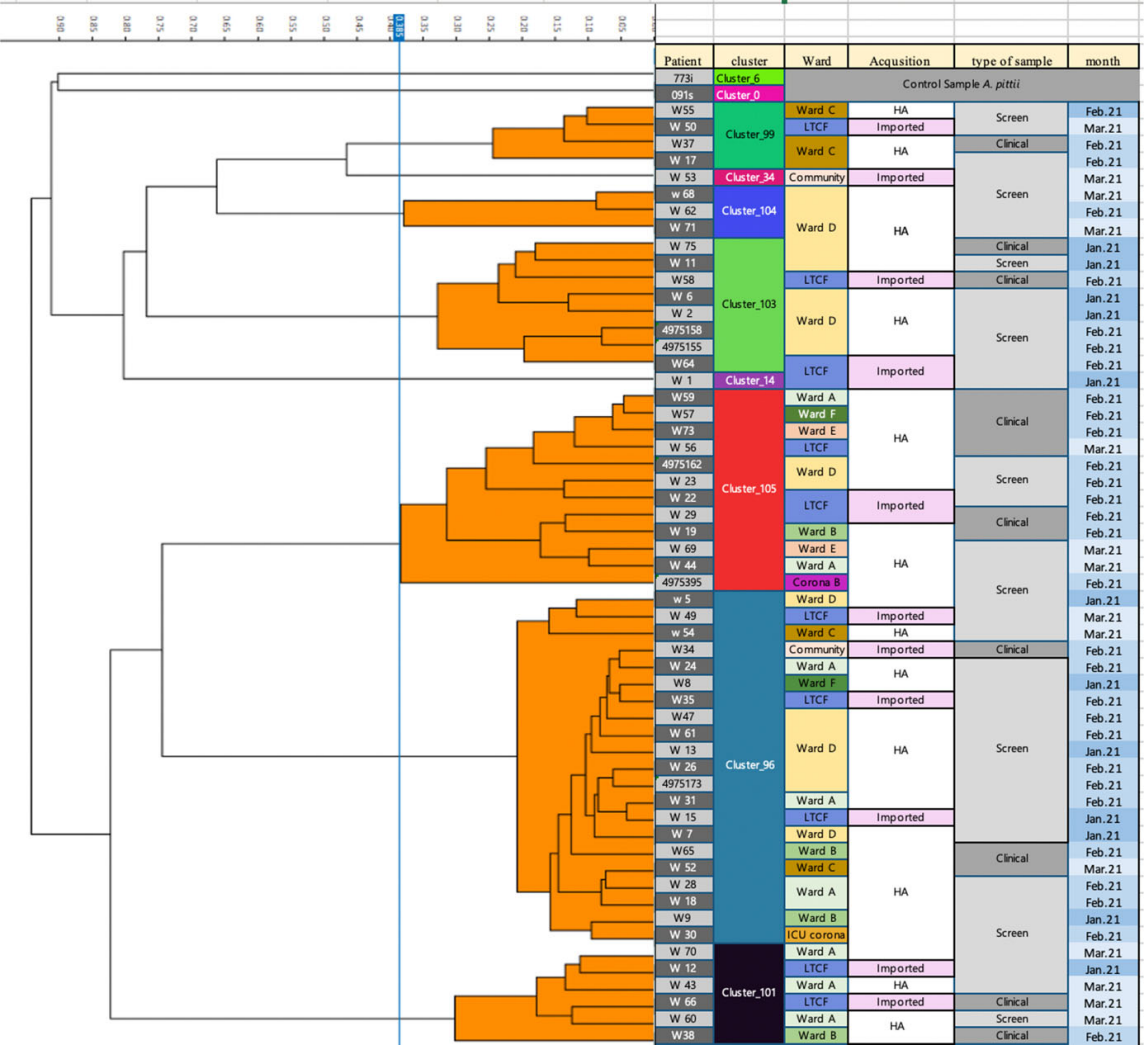
Dendrogram carbapenem-resistant *A. baumannii* isolates obtained by Fourier transform infrared biotyping. The dendrogram analysis reveals that multiple clusters were simultaneously disseminated within the hospital and within the same ward. Screening samples and imported cases were involved in the spread of these clusters. Note. HA, hospital acquired.

From January 2021 to March 2021, 55 patients were detected with CRAB. Of these, 23.6% were identified during the first 3 days of hospitalization. There were 14 clinical isolates and 41 screening cultures. All samples were available for phenotyping using the FTIR Biotyper. We identified 8 distinct clones (Fig. 3). Spatiotemporal overlap was detected in 7 distinct transmission episodes involving 31 patients (56%). Also, 3 transmission episodes with 12 hospital-acquired cases were related to 4 imported cases.

### Active surveillance program

**Figure 4.**
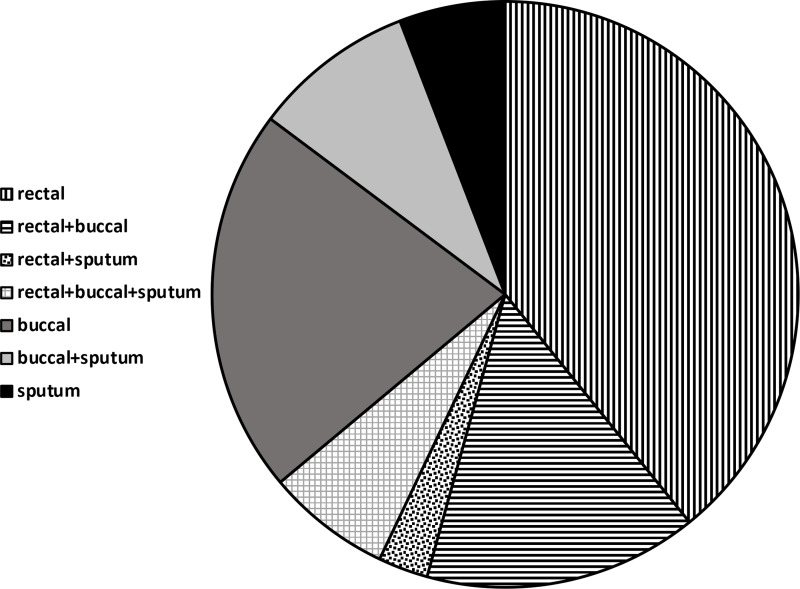
Site of carbapenem-resistant *Acinetobacter baumannii* carriage among patients detected by screening cultures.

**Figure 5.**
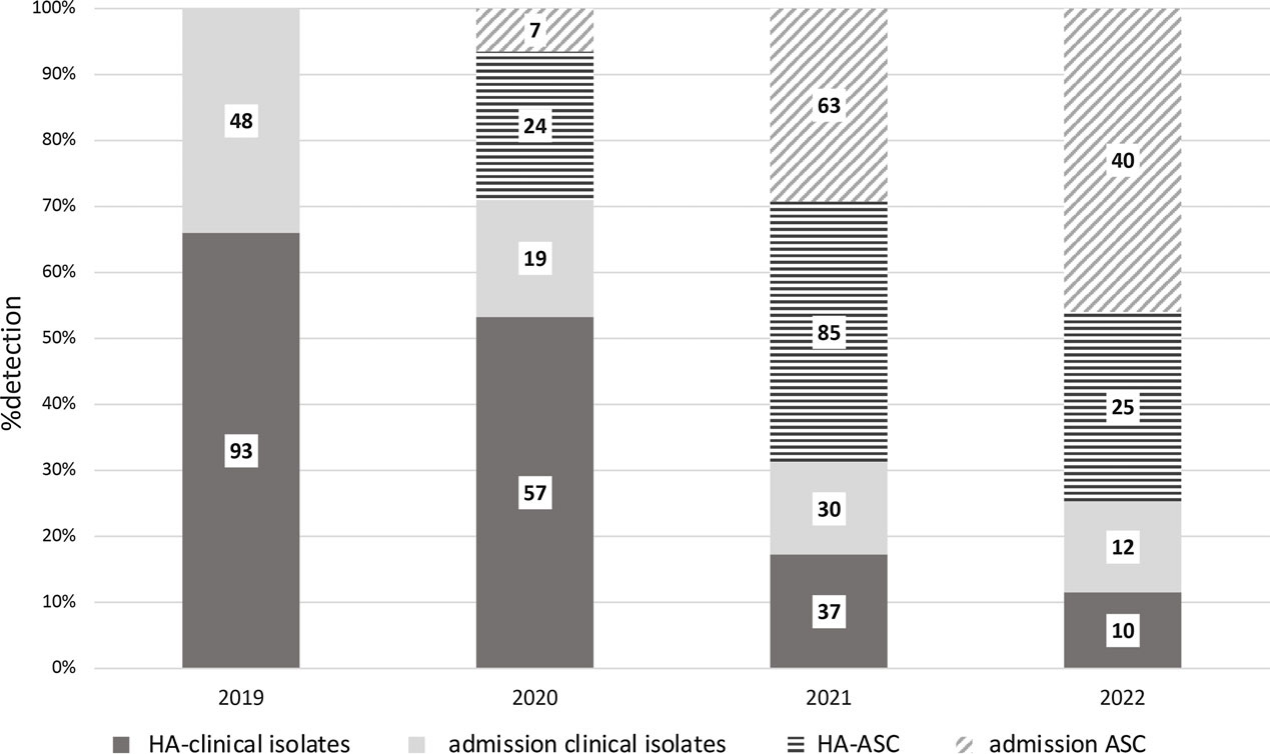
Mode of initial detection of carbapenem-resistant *Acinetobacter baumanni*, 2019–2022. Note. CRAB, carbapenem-resistant *Acinetobacter baumannii*; HA, hospital acquired. The numbers within the column indicate the total count of cases.

From June 2020 to September 2022, a total of 23,626 screening cultures were obtained. The number of surveillance cultures increased from a monthly average of 195.4 (SD±119.9) during phase II to 874.0 (SD±79.7) during phase III and 1,107 (SD±124) during phase IV. An average of 1.8 sites (SD±0.37) were obtained from each patient. Figure 4 describes the contribution of each site. The combination of rectal and buccal sites detected 90.6% of carriers. During the same period, 410 patients were identified as being colonized or infected with CRAB. Of these 410 patients, 266 (64.9%) were initially identified by screening, and 144 (35.1%) were identified by clinical microbiologic cultures. CRAB was detected solely by active screening cultures in 190 patients, accounting for 46.3% of all CRAB isolates. Among the 266 carriers, 77 patients (28.6%) were subsequently identified as having positive CRAB growth in clinical cultures; the most common site was sputum (58.4%), followed by wound (29.9%) and blood (16.7%). The median duration between initial surveillance culture and subsequent clinical sample was 16 days (IQR, 4–32). Linear regression analysis revealed a statistically significant increase in the proportion of CRAB detections identified through screening from 29.0% (31/107) in 2020 to 76.3% (65/87) in 2022 (*P* < .001). A significant increase was also observed in the proportion of imported cases, rising from 24.3% (26/107) in 2020 to 61.0% (52/87) in 2022. (*P* < .001) (Fig. 5).

Among the 2,519 patients screened after day 3 of hospitalization, 143 (5.7%) were found to be colonized, with a median stay of 13 days (IQR, 7–26) before detection. The rate of newly detected carriage among patients screened weekly decreased significantly from 6.5% (85 out of 1,299) in 2021 to 3.7% (26 out of 694) in 2022 (*P* < .01). No significant difference in carriage rates was observed among patients screened upon admission during these years, with rates of with rates of 2.2% (63/2,832) in 2021 and 2.8% (40/1,426) in 2022 (*P* = .43).

Pearson correlation coefficients between clinical CRAB IDR and various interventions were calculated. A moderate correlation was found between clinical CRAB rates and low rates of carriage among patients screened weekly (Pearson correlation, 0.55; *P* = .04). No significant correlation was found with the percentage of cohorting (Pearson correlation, 0.219; *P* = .264) nor cleaning quality (Pearson correlation, 0.209; *P* = .29).

## Discussion

In recent years, CRAB has become a significant nosocomial pathogen, leading to increased patient morbidity, mortality, and healthcare costs.^20^ To combat CRAB dissemination in healthcare facilities, effective control measures are crucial. We implemented a multifaceted intervention, including cohort placement of infected patients, dedicated nursing, intensified cleaning, hand hygiene monitoring, and small-scale screening. Despite high compliance with these measures, CRAB continued to spread in several wards. We then introduced a large-scale screening program on admission and weekly in step-up units and ICUs, resulting in a hospital-wide decrease in HA-CRAB rates.

The importance of active screening in controlling CPE is widely acknowledged and recommended by guidelines.^7,15^ In contrast, its role in CRAB control remains debated. Previous studies in endemic settings used active screening in multifaceted interventions, making it hard to determine the specific contribution of the active screening.^13,14^ In the current study, 64.9% of CRAB carriers were initially identified via active screening cultures, with approximately 50% of patients identified exclusively through screeing cultures. Decreased clinical HA-CRAB rates was associated with reduced nosocomial cases detected by weekly screening. FTIR analysis showed the presence of 8 clones, with imported and hospital-acquired strains closely related, highlighting the role of imported CRAB carriers in the persistence of CRAB in endemic healthcare settings.

Surveillance sites, the number of samples, frequency of periodic screening, and the microbiologic methods used may affect the detection of a CRAB carrier.^21^ In contrast to CPE detection, where rectal sampling is sufficient, sampling multiple sites is required to detect CRAB carriers. Previous experts have suggested the culture of multiple patient sites.^15^ However, this approach may impose a significant workload both on nursing and laboratory personnel. In a recent review, the best performance was obtained by culturing skin (100%), followed by rectal samples (86%).^22^ Although our intervention did not include skin samples, high sensitivity for detecting CRAB carriers can be achieved with rectal and pharyngeal swabs.^23^ Previous studies have also reported persistent decreases in CRAB rates using surveillance screening without skin swabs.^14,24^

The COVID-19 pandemic has led to a significant increase in hospital-acquired MDRO rates in many regions.^25^ According to the CDC, there has been a 78% increase in hospital-onset CRAB cases.^26^ This increase is likely due to a variety of factors, including human factors such as staffing shortages, inadequate use of personal protective equipment, and poor hand hygiene compliance. Notably, we did not find a correlation between CRAB rates and periods of peak COVID-19 hospitalizations (data not shown). CRAB rates decreased despite reduced hand hygiene compliance. Previous studies have also reported decreases in hand hygiene compliance during the late stages the COVID-19 pandemic era.^27,28^ Early detection of carriers and cohorting with dedicated nursing may compensate for the suboptimal hand hygiene compliance.

Interventions in the literature primarily address outbreaks in ICUs, with limited data on comprehensive strategies in endemic non-ICU areas.^13,29,30^ Data describing comprehensive interventions in endemic non-ICU areas are limited. We faced hospital-wide spread of CRAB, predominantly in 6 open-space step-up units in medical wards. Notably, most acute-care hospitals in Israel have insufficient ICU beds. As a result, many patients are mechanically ventilated in internal medicine wards.^31^ The presence of ventilated patients in medical departments generates clinical and infection control challenges, including increased risk for the emergence and spread of MDROs. Although contact precautions and single-room placement are crucial measures of MDRO control,^8^ they were unattainable for >50% of CRAB-infected patients during the baseline phase. Dedicated HCWs may reduce the likelihood of transmission of bacteria from a colonized patient to an uncolonized patient.^32–34^ Therefore, the initial control measures were focused on placing carriers in cohorts in an isolation ward with dedicated nursing care. The impact of these interventions was possibly limited due to a sizable influx of unknown CRAB carriers.

This study had several limitations. The lack of a control population and the presence of concurrent infection control measures made it difficult to ascertain whether the active screening program was the key factor in the decrease of CRAB rates or whether other control measures or random variation contributed. Additionally, the study was conducted in a single center with specific setting characteristics, such as the design of open, multibed rooms with closely spaced beds. Therefore, these findings may not be applicable in hospitals with different infrastructures. Data on compliance with obtaining active screening cultures were not collected due to limitations of the electronic medical record, but efforts were made to improve compliance through weekly reminders and manual evaluation by infection control preventionists. Lastly, the study was conducted during different phases of the COVID-19 pandemic, which could have affected healthcare-worker compliance with infection control measures and the colonization rate of CRAB due to changes in hospitalization patterns of patients with and without COVID-19.

In conclusion, a multifaceted intervention resulted in a persistent decrease in the rate of HA-CRAB. Sizable proportions of both the imported and HA-CRAB cases were detected by screening cultures. The use of surveillance cultures to identify the population of asymptomatic carriers may enhance the effectiveness of a CRAB infection prevention programs in endemic settings.
